# Tumor and Stromal Sphingolipid Imbalance Are Associated with T Cell Tissue Residency in Glioblastoma

**DOI:** 10.3390/cancers18132168

**Published:** 2026-07-06

**Authors:** Chase M. Walton, Elif Percin, Han Gyul Lee, Odai Darawsha, Ben A. Strickland, Besim Ogretmen

**Affiliations:** 1 Department of Biochemistry and Molecular Biology, Medical University of South Carolina, Charleston, SC 29425, USA; waltonch@musc.edu (C.M.W.); percin@musc.edu (E.P.); leehan@musc.edu (H.G.L.); darawsha@musc.edu (O.D.); 2 Hollings Cancer Center, Medical University of South Carolina, Charleston, SC 29425, USA; strickbe@musc.edu; 3 Department of Neurosurgery, Medical University of South Carolina, Charleston, SC 29425, USA

**Keywords:** glioblastoma, melanoma, tumor-infiltrating lymphocytes, tissue-resident memory, sphingosine-1-phosphate, ceramide, single-cell RNA sequencing

## Abstract

Background/Objectives: T cells within solid tumors often switch from a recirculating to a tissue-resident state, which may blunt antitumor activity, but the signal driving this switch in vivo remains unclear. We asked whether alterations in sphingosine-1-phosphate (S1P) signaling in tumor or the surrounding stromal cells are associated with T cell residency in glioblastoma (GBM). Methods: We analyzed five single-cell RNA-sequencing cohorts: three human glioma datasets, an in-house mouse CT2A glioblastoma cohort, and a human melanoma tumor-infiltrating lymphocyte cohort. T cell egress and tissue-residency programs, together with stromal S1P production and degradation, were scored per cell using curated gene modules. Cell-state contrasts were quantified as Cohen’s d, and sample-level coupling as Spearman ρ. Results: In human GBM, T cell residency programs were elevated in CD4+ helper and regulatory T cells in tumors compared with low-grade glioma controls. In mouse CT2A-derived GBM tumors, stromal S1P production correlated negatively with T cell residency across four independent stromal cell types. In human GBM microglia, S1P production was reduced compared with control microglia. The same CD8+ residency phenotype was replicated in CD3-sorted GBM tumor-infiltrating lymphocytes (TILs) and in melanoma TILs. Conclusions: A loss of stromal S1P production accompanies T cell tissue residency in GBM. Thus, stromal S1P metabolism is a candidate axis for modulating T cell recirculation and TIL biology in GBM. These findings are transcriptomic associations from single-cell RNA sequencing that do not directly measure S1P metabolite levels or signaling activity and will require functional and lipidomic validation.

## Simple Summary

Tumor-infiltrating T cells often lose the ability to leave the tumor and instead acquire a tissue-resident phenotype that may blunt their effectiveness against cancer. What causes them to enter this state in vivo has not been fully resolved. Using single-cell RNA-sequencing data from human and mouse glioblastoma tissues, we found that these T cells switch off the genes that allow them to re-enter the circulation and switch on the genes that keep them in place. The shift tracks with the surrounding stromal cells losing their ability to produce sphingosine-1-phosphate (S1P), a bioactive lipid that normally guides T cells back into the blood and lymph. The same pattern holds in T cells from melanoma tumors. A shared stromal loss of S1P production, therefore, tracks with the residency state of T cells across two different solid tumors.

## 1. Introduction

Glioblastoma (GBM) is the most common and most aggressive primary brain malignancy in adults. Even after the standard of care of maximal safe resection, radiation, and concurrent temozolomide [1], recurrence is uniform, and median survival for IDH (isocitrate dehydrogenase)-wild-type disease remains under two years [1,2]. It accounts for roughly half of all malignant primary brain tumors and carries among the poorest survival of any human cancer [3,4], and its IDH-wildtype definition under the WHO CNS5 classification underscores a molecularly distinct and uniformly lethal entity [5]. A central barrier is its immunologically “cold,” immunosuppressive microenvironment, which is dominated by tumor-associated myeloid cells and constrains effective T cell immunity [6,7,8]. T cell infiltration of solid tumors is a defining feature of the antitumor immune response, and single-cell RNA sequencing studies have revised the textbook picture of which T-cell state populate tumor tissue. Many solid tumors harbor substantial T cell infiltration [9], yet GBM-infiltrating T cells display exhaustion programs that limit antitumor function [10,11,12]. Checkpoint blockade has not delivered the benefit in unselected recurrent GBM [13] that was anticipated from neoadjuvant studies showing intratumoral and systemic immune responses [14]. In GBM and other advanced solid tumors, the infiltrating T cells exhibit a tissue-resident memory (TRM) phenotype rather than a circulating effector phenotype [15,16]. In addition, GBM patients show striking sequestration of naïve T cells in the bone marrow rather than in the circulation [17]. Across solid tumors, the density of CD8+ TRM cells tracks with favorable outcome and improved response to immunotherapy [18,19], while treatment-induced lymphopenia is associated with worse survival in GBM [20], making the trafficking decisions that govern TIL retention versus recirculation clinically consequential. Whether alterations in these trafficking decisions underlie the tumor-infiltrating lymphocyte (TIL) TRM phenotype remains largely unknown.

T cell TRM phenotype is governed primarily by the sphingosine-1-phosphate (S1P) and S1P receptor 1 (S1PR1) axis [21]. T cells expressing surface S1PR1, KLF2, and the lymph-circulation markers such as SELL and CCR7 exit tissue by following the S1P gradient back to blood and lymph—the recirculating effector phenotype. The TRM T cells, by contrast, downregulate this egress program and acquire markers such as CD69, ITGAE, ZNF683 (Hobit), RUNX3, and CXCR6 [22,23]. The transition is pharmacologically tractable: fingolimod (FTY720) blocks S1PR1 and sequester T cells in lymphoid tissues [24]. More broadly, S1P-receptor signaling is a master regulator of immune-cell trafficking and a tractable therapeutic node [25], and sphingosine kinase activity has been linked to immunosuppressive myeloid programs in glioma [26].

Less attention has been paid to the upstream determinants of local S1P accumulation and metabolism in tumor stroma. Stromal cells of the tumor microenvironment (endothelium, pericytes, astrocytes, and tissue-resident myeloid cells) express the sphingosine kinases 1 and 2 (SPHK1 and SPHK2) that synthesize S1P, as well as SGPL1 (S1P lyase), which degrades it. Many of these same cells also express ceramide synthases 1-6 (CERS1-6 family) and SMPD3 (sphingomyelinase) on the opposing arm of the pathway. Sphingolipid metabolism is established as a regulator of cancer cell survival, proliferation, and stress response, with elevated S1P signaling and altered ceramide handling implicated in malignant progression [12,27,28,29,30]. Sphingolipids are also active regulators of inflammatory signaling more broadly [31]. Within the tumor immune compartment, ceramide and S1P-axis enzymes have been directly tied to T cell function: ceramide synthesis controls antitumor T cell metabolism and mitochondrial fitness [12], ceramide-synthase activity regulates PD-L1 trafficking and Treg infiltration in solid tumors [30], and pharmacological inhibition of sphingosine kinase 2 enhances antitumor T cell activity and synergizes with immunotherapy in preclinical models [32]. Whether tumors alter this stromal sphingolipid balance in vivo, and whether such alterations track with local T cell residency in GBM, has not been examined systematically.

To address this question, we analyzed five single-cell RNA-sequencing cohorts spanning human GBM discovery, an enriched human GBM TIL cohort, human GBM microglia, and mouse CT2A-derived GBM, and compared them with human melanoma TILs. We asked whether a shared stromal sphingolipid signal accompanies T cell residency across these cohorts. To our knowledge, this is the first systematic multi-cohort single-cell association of stromal (rather than T-cell-intrinsic) S1P-production loss with the T cell TRM phenotype in GBM, resolved across individual stromal cell types (myeloid, pericyte, endothelial, astrocyte, and microglia) and conserved across species (human and mouse) and tumor types (GBM and melanoma). This complements prior work localizing T-cell-intrinsic S1PR1 disruption to the bone marrow [17] by implicating a distinct anatomical station, the tumor stroma. As a purely transcriptomic, associational analysis without direct S1P measurement or functional validation, our findings nominate stromal S1P metabolism as a candidate axis and T cell TRM as a candidate readout of impaired TIL recirculation, to be tested in future mechanistic studies.

## 2. Materials and Methods

### 2.1. Single-Cell RNA-Sequencing Cohorts

#### 2.1.1. Public Single-Cell Cohorts

Four publicly accessible single-cell datasets were assembled in addition to the in-house mouse data. Three human glioma cohorts were used. Abdelfattah 2022 (GSE182109) [33]: *n* = 13 donors, 250,955 cells spanning low-grade glioma (LGG) controls, primary GBM, and recurrent GBM. Mathewson 2021 (GSE163108) [34]: *n* = 5 patients, 25,256 CD3-sorted T cells with TCR clonotypes. Sankowski 2019 (GSE135437) [35]: *n* = 65 samples, 12,672 CD45 + CD11b+-sorted microglia (7849 passing quality control across the GBM and control donor groups analyzed here). The cross-cancer cohort was Sade-Feldman 2018 (GSE120575) [36]: *n* = 16,291 melanoma TILs with pre- and post-immunotherapy timepoints.

#### 2.1.2. In-House Mouse CT2A scRNA-Seq

In-house mouse glioblastoma data were generated in the Strickland laboratory using the orthotopic CT2A model. Wild-type male C57BL/6J mice (8–12 weeks at implantation) received intracranial implantation of 10,000 CT2A cells in 2 μL via stereotactic injection into the right striatum (AP +0.5, ML +2.0, DV −3.0 mm from bregma) using a Hamilton syringe (Hamilton Company, Reno, NV, USA) at a slow infusion rate; tumors were harvested 21 days post-implantation. Single-cell suspensions were prepared using the Miltenyi Biotec Tumor Dissociation Kit (Miltenyi Biotec, Auburn, CA, USA) (mouse, #130-096-730) on a gentleMACS Octo Dissociator (Miltenyi Biotec, Auburn, CA, USA) with Heaters per manufacturer instructions, followed by Miltenyi Debris Removal Solution (Miltenyi Biotec, Auburn, CA, USA) (#130-109-398) and Dead Cell Removal Kit (Miltenyi Biotec, Auburn, CA, USA) (#130-090-101) per manufacturer protocols.

Single-cell libraries were prepared using the 10× Genomics Chromium Next GEM Single Cell 3′ Kit v4 (10× Genomics, Pleasanton, CA, USA), targeting approximately 20,000 cells per channel. Libraries were submitted to Azenta Life Sciences (GENEWIZ; South Plainfield, NJ, USA) for sequencing on an Illumina NovaSeq instrument (Illumina, Inc., San Diego, CA, USA) at the manufacturer-recommended depth for v4 chemistry (~25,000 reads per cell). Three sequencing batches (DS1, DS2, DS3) were processed with Cell Ranger version 7 (10× Genomics; Pleasanton, CA, USA); DS3 additionally included paired central and peripheral tumor samples per mouse. Batches were integrated into a unified object using scVI (version 1.4.2) [37]. For the analyses presented in this work, the control and treatment arms of the integrated mouse object were analyzed together, with the different treatment arms serving as inter-treatment controls. Downstream analysis was performed using Scanpy (version 1.12) [38]. Standard quality control was applied: cells with fewer than 200 detected genes or greater than 10% mitochondrial reads were excluded.

Cell-type annotations were used as provided by the source publication for each public cohort, with no re-clustering performed within this work except where explicitly noted. Source-author annotations and their underlying identification strategies were as follows. Abdelfattah 2022 [33]: Seurat clustering of the integrated GSE182109 object with broad categories of T cells (*n* = 26,850), Myeloid (*n* = 110,295), and Other (*n* = 113,810). Mathewson 2021 [34]: CD3+ FACS sorting prior to scRNA-seq, with T-cell state and subtype annotations from source-author clustering (non-T-cell types not present by design). Sankowski 2019 [35]: CD45 + CD11b+ microglia FACS sorting prior to scRNA-seq (only microglia compartment present). Sade-Feldman 2018 [36]: T-cell state annotations from source-author clustering. For the in-house mouse CT2A object, cell-type labels were assigned using canonical marker panels in the Strickland lab Scanpy/scVI annotation pipeline: Cd45 for immune cells, Cd68/Mrc1 for myeloid (bone marrow-derived macrophage, BMDM), Tmem119/P2ry12 for microglia, Pecam1/Cldn5 for endothelial, Pdgfrb for pericyte, Gfap/Aqp4 for astrocyte, Plp1/Mog for oligodendrocyte, Cd3e/Cd3d for T cell, with residual malignancy-marker-positive cells labeled Tumor.

For the compartment-resolved analysis presented in Section 3.4 below, the Abdelfattah 2022 [33] ‘Other’ cluster (*n* = 113,810 cells) was subdivided into Tumor versus Other nontumor by scoring each cell on a malignancy marker module (SOX2, EGFR, OLIG1, OLIG2, GFAP, VIM, NES, FABP7, MKI67; markers established for GBM in Abdelfattah 2022 [33]) together with a normal-neural counter module (RBFOX3, NEFL, SYN1, SYP, MOG, MBP) to suppress false positives from incidental marker overlap in mature neurons and oligodendrocytes. Cells with a malignancy score greater than zero and a normal-neural score less than or equal to zero were reclassified as Tumor (*n* = 78,149 of 113,810); the remaining 35,661 cells were left labeled Other. This re-annotation is documented in the analysis repository at reannotate_gse182109_tumor.py and is the only re-clustering/re-labeling step performed on any public cohort in this work. The re-annotation threshold space and the resulting final cell-type counts are shown in Supplementary Figure S1. Per-cohort cell-type composition by sample is provided in Supplementary Figure S2A (GSE182109) and S2B (mouse CT2A). This re-annotation was applied solely to subdivide the source-author ‘Other’ cluster for the descriptive, compartment-level gene expression heat maps; it does not enter the quantitative T cell residency or stromal-coupling analyses, which operate on the independently annotated T cell and stromal compartments. The principal findings are therefore insensitive to the re-annotation threshold.

### 2.2. Gene Module Definitions

Hypothesis modules were curated from the canonical sphingolipid-pathway and tissue-residency literature, then pruned based on expression diagnostics in mouse CT2A T cells (genes detected in fewer than 1% of T cells were dropped). Pruning genes below this detection floor avoids scoring being dominated by sparse, near-zero counts that contribute noise rather than signal in single-cell data. The pruned T cell egress module (human) comprised S1PR1, KLF2, SELL, LEF1, TCF7 (CCR7, although canonical, fell below the 1% detection threshold in mouse CT2A T cells and was pruned from the scored module). The T cell tissue-resident memory module comprised CD69, ITGAE, ITGA1, ZNF683, RUNX3, CXCR6, and PRDM1. The egress module captures the transcriptional program enabling T cell recirculation out of tissue, combining the S1P receptor that senses the S1P gradient (S1PR1), its transcriptional regulator (KLF2), and lymph-node-homing/naive-like markers (SELL, LEF1, TCF7). The residency module captures the reciprocal program that retains T cells in tissue, spanning early activation/retention markers (CD69, which antagonizes S1PR1), integrins anchoring cells to the parenchyma (ITGAE, ITGA1), residency-defining transcription factors (ZNF683, RUNX3, PRDM1), and the chemokine receptor that supports tissue positioning (CXCR6). The local S1P synthesis and accumulation axis comprised SPHK1, SPHK2, SPNS2, ABCC1, MFSD2B. The local S1P degradation axis comprised SGPL1, PLPP1, PLPP2, PLPP3. The synthesis/accumulation axis aggregates the kinases that synthesize S1P (SPHK1, SPHK2) and the transporters that export and accumulate it extracellularly (SPNS2, ABCC1, MFSD2B), whereas the degradation axis aggregates the lyase that irreversibly cleaves S1P (SGPL1) and the phosphatases that dephosphorylate it back to sphingosine (PLPP1, PLPP2, PLPP3); together these proxy the capacity of a cell to raise versus lower local S1P availability. Mouse orthologs were used for mouse cohorts. The S1P balance composite was computed per cell as local S1P production minus local S1P degradation. A positive balance score thus indicates a transcriptional program tilted toward generating and accumulating local S1P, and a negative score indicates one tilted toward clearing it. These modules are transcriptional proxies for pathway activity, reflecting enzyme, transporter, and marker mRNA abundance scored against expression-matched control gene sets [39]; they do not directly measure S1P metabolite levels, S1P flux, or S1PR1 signaling activity, and should be interpreted accordingly. The choice of these genes follows the canonical S1P-metabolism literature, in which the sphingosine kinases SPHK1 and SPHK2 together with the SPNS2, ABCC1, and MFSD2B transporters set local S1P production and export, while SGPL1 and the PLPP phosphatases set its degradation [40]; SPHK transcript abundance has been reported to track measured S1P levels in human tumor tissue [41], and gene-set scoring is an established approach for inferring pathway activity from single-cell transcriptomes [42]. Even so, enzyme and transporter transcript abundance is an imperfect surrogate for metabolite flux, because post-transcriptional regulation, enzyme localization, and substrate availability all shape actual S1P levels; these module scores are therefore best read as candidate indicators of pathway tone that orthogonal lipidomics would be needed to confirm.

For figures, gene-level heat maps additionally display an expanded ceramide-axis panel (CERS1, CERS2, CERS4, CERS5, CERS6, SMPD1, SMPD2, SMPD3, DEGS1, ASAH1, ASAH2, ACER2, ACER3) and the full S1P-axis enzyme panel (production and degradation enzymes). Heat map values are mean log-normalized expression per cell type or stratum, with rows z-scored for visualization.

### 2.3. Module Scoring and Statistical Analysis

Module scores were computed with scanpy.tl.score_genes [38] using default control gene sampling (ctrl_size = 50, n_bins = 25) against log-normalized count matrices. The underlying gene-set scoring approach follows Tirosh et al. [39] by matching each signature gene to control genes in a comparable expression bin. Effect sizes for cell-state and disease contrasts were quantified as Cohen’s d, with 95% confidence intervals derived from the standard small-sample variance approximation. Donor-level analyses aggregated per-cell scores to per-donor means before computing Spearman correlations or Mann–Whitney U tests. Multi-cohort integration of human cohorts at the per-cell level was not attempted; each cohort was analyzed independently, following standard single-cell analysis practices [43]. All statistical tests were two-tailed. Within each figure that involves multiple Spearman or Mann–Whitney tests across cell types or strata, *p*-values were corrected using the Benjamini–Hochberg false discovery rate procedure, and q-values are reported alongside *p*-values in the corresponding Results sentences. Inference is led by effect size (Cohen’s d for cell-state contrasts; Spearman ρ for sample-level coupling) and by replication across independent cohorts.

### 2.4. Code and Data Availability

All analysis code is available at https://github.com/chasewalton/stromal-sphingolipid-trm-axis (accessed on 4 May 2026). Processed single-cell objects for the integrated mouse CT2A cohort, along with per-cell module scores for all five cohorts, will be deposited at Zenodo prior to publication. Per-figure result tables, including effect sizes, sample sizes, and statistical test outputs, are provided as Supplementary Tables S1–S5. No generative artificial intelligence was used to generate scientific data, results, or interpretations; an AI coding assistant was used for software drafting and statistical scripting under direct author review, as disclosed in the Acknowledgments.

## 3. Results

### 3.1. T Cell Egress and Residency Programs Shift Together in Human GBM

**Figure 1:**
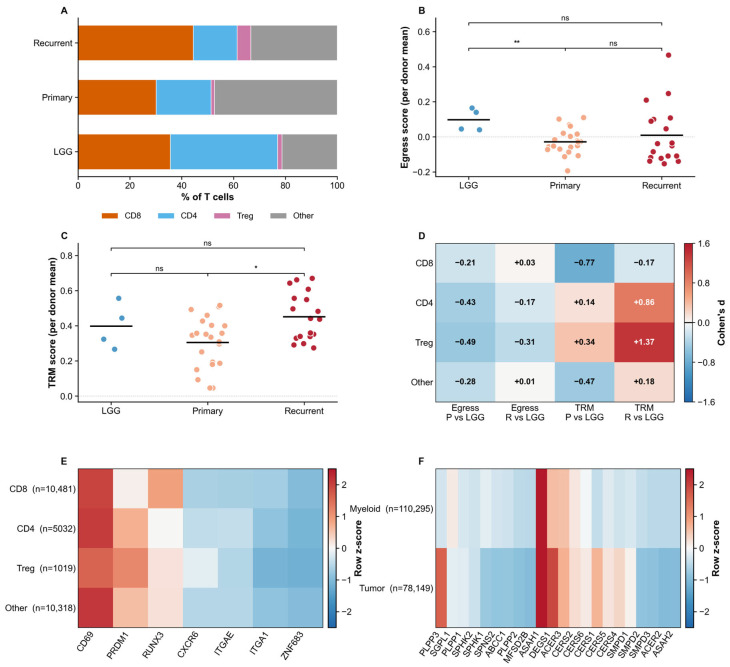
Discovery of the T cell tissue-residency signature in human glioblastoma (GSE182109; *n* = 13 donors, 26,850 T cells). (**A**) T cell subset composition (CD8, CD4, Treg, Other) by tumor stage. (**B**) Per-donor mean egress score (S1PR1, KLF2, SELL, LEF1, TCF7 module) by tumor stage with Mann–Whitney brackets. (**C**) Per-donor mean TRM score (CD69, ITGAE, ITGA1, ZNF683, RUNX3, CXCR6, PRDM1) by tumor stage. (**D**) Per-subset Cohen’s d heat map for module scores (Primary vs. LGG and Recurrent vs. LGG). (**E**) TRM module gene expression heat map (mean log1p per T cell subset; row z-score). (**F**) Stromal S1P + ceramide-axis gene heat map (mean log1p per cell type; row z-score). Abbreviations: TRM, tissue-resident memory; LGG, low-grade glioma; S1P, sphingosine-1-phosphate. Box plots (**B**,**C**) show the median (center line), interquartile range (box) and whiskers; each point is one donor (*n* = 13). Brackets denote two-tailed Mann–Whitney U tests (significance at *p* < 0.05). Panels (**E**,**F**) are descriptive expression maps (no disease test): “mean log1p” is the mean log1p-normalized counts per group and “row z-score” standardizes each gene row across columns. Overall, panels (**A**–**D**) establish the human GBM T cell tissue-residency signature and show that it is concentrated in CD4+ helper and regulatory T cells, whereas the CD8+ contrast is underpowered by the small LGG control population; panels (**E**,**F**) map the underlying T-cell and stromal gene expression that motivates the cross-cohort analyses in Figure 2, Figure 3 and Figure 4. * *p* < 0.05, ** *p* < 0.01.

To determine whether T cell egress and residency programs shift together in human GBM, we first evaluated pan-T cell module scores in a cohort of 13 patients spanning LGG controls, primary GBM, and recurrent GBM (GSE182109, Abdelfattah 2022 [33]). T cell subset composition by tumor stage and module score distributions for each subset are shown in Figure 1A–C and in Supplementary Figures S1 and S2. At the pan-T cell level, the egress program decreased, and the residency program increased in tumor versus control, but the shifts were modest at this aggregate level (Cohen’s d for egress = −0.12, TRM = +0.18 with pruned modules). The donor-level TRM mean was significant only across the three groups taken together (Kruskal–Wallis *p* = 0.032, *n* = 13). The signal is concentrated in CD4+ helpers and regulatory T cells (Tregs) rather than in the CD8+ majority: the subset-resolved Cohen’s d heat map (Figure 1D; Supplementary Table S1) shows TRM gains of d = +0.86 in CD4+ and d = +1.37 in Tregs in the Recurrent-vs-LGG contrast, with the CD8+ contrast underpowered (d = −0.17) because only 85 LGG CD8+ cells were available. In Figure 1D, rows are T cell subsets (CD8/CD4/Treg/Other), and columns are the four disease contrasts (Egress Primary vs. LGG, Egress Recurrent vs. LGG, TRM Primary vs. LGG, TRM Recurrent vs. LGG); cell values are Cohen’s d with white text marking |d| ≥ 0.3. Per-subset expression of the seven canonical TRM genes (CD69, PRDM1, RUNX3, CXCR6, ITGAE, ITGA1, ZNF683) is shown in Figure 1E, and the per-compartment baseline expression of the stromal S1P-axis and ceramide-axis enzymes in myeloid versus tumor cells is shown in Figure 1F. S1PR1 is reported only as a single gene within the composite “T cell egress” and is never reported on its own due to low detection in this dataset (≈5% of T cells). Figure 1E,F are descriptive expression maps with no disease contrast; the disease contrast is shown in Figure 1D.

The CD8+ underpower visible in Figure 1D motivated a power-rescue analysis in a separate cohort. We evaluated the Mathewson 2021 [34] cohort of CD3-enriched GBM TILs (21,502 CD8+, 3277 CD4+, and 388 Treg cells, plus 89 cells in other states; 25,256 total), in which the CD8+ versus CD4+ contrast cleared the |d| ≥ 0.3 threshold in both modules (egress d = −0.35, TRM d = +0.36; see Section 3.4 below). These data indicate that the CD8+ residency program is reproducibly observed in human GBM TILs. The GSE182109 CD8+ contrast was attenuated by the size of the LGG control population, not by an absence of underlying biology. Confidence intervals for all per-subset effect sizes underlying the Figure 1D heat map are provided as a forest plot in Supplementary Figure S3A–D, including per-cell *n* for each row.

### 3.2. Low Stromal S1P Production Tracks with T Cell Residency in Mouse Glioblastoma

**Figure 2:**
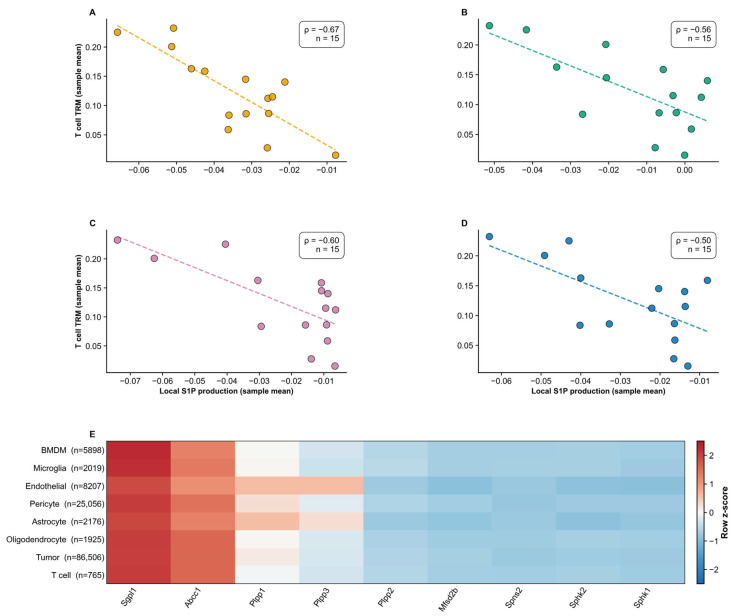
Stromal local S1P production is inversely associated with T cell TRM scores in mouse CT2A glioblastoma control tumors. (**A**–**D**) Per-sample (*n* = 15 mouse samples) scatter of stromal local S1P production score against T cell TRM score for four stromal cell types: (**A**) BMDM, (**B**) endothelial, (**C**) pericyte, and (**D**) astrocyte. Each point represents one mouse sample. Dashed line shows the linear regression fit, truncated at observed data bounds. Spearman ρ and sample size are annotated in the upper-right of each panel. Panel titles in red indicate cell types passing the |ρ| ≥ 0.3 and *p* < 0.05 thresholds. (**E**) Compartment-resolved S1P-axis gene expression heat map in control tumors (rows = six stromal cell types + Tumor + T cell; mean log1p, row z-score). The 8-row layout directly tests whether S1P-axis enzyme transcripts partition to stromal compartments or are shared with tumor cells and T cells. Abbreviation: BMDM, bone marrow-derived macrophage. Correlations in (**A**–**D**) are two-tailed Spearman ρ. Panel (**E**) is a descriptive expression map (no disease test): mean log1p-normalized expression, row z-scored across columns. Overall, panels (**A**–**D**) show that lower stromal local S1P production co-occurs with higher T cell tissue residency across four independent mouse stromal cell types, and panel (**E**) shows that the production-arm enzymes (Sphk1, Sphk2, Spns2) and the degradation-arm enzyme Sgpl1 partition reciprocally across these stromal compartments, anchoring the module-level couplings in panels (**A**–**D**).

To assess whether the T cell phenotype is associated with a coordinated stromal signal, we examined sample-level concordance between stromal sphingolipid scores and T cell TRM scores in the in-house mouse CT2A-derived GBM versus control cohort (*n* = 15 samples). Stromal local S1P production scores correlated negatively with T cell TRM scores across multiple stromal compartments: BMDM Spearman ρ = −0.67 (*p* = 0.007, Benjamini–Hochberg q = 0.033), endothelial ρ = −0.56 (*p* = 0.030, q = 0.050), pericyte ρ = −0.60 (*p* = 0.017, q = 0.043), and astrocyte ρ = −0.50 (*p* = 0.058, q = 0.072, borderline) (Figure 2A–D). The q-values reflect Benjamini–Hochberg correction across the five stromal cell types tested for sample-level coupling (BMDM, endothelial, pericyte, astrocyte, and oligodendrocyte); the fifth type, oligodendrocyte, showed a weak positive, non-significant coupling (ρ = +0.10, *p* = 0.72, q = 0.72). In each panel of Figure 2A–D, the *x*-axis is the local S1P production score (sample mean) for one stromal cell type and the *y*-axis is the T cell TRM score (sample mean); each point represents one of *n* = 15 mouse CT2A samples (Supplementary Table S2). The four panels share identical axes and differ only in which stromal cell type provides the *x*-axis score (A: BMDM; B: endothelial; C: pericyte; D: astrocyte). The consistent direction across four independent stromal cell types, three at or below the q = 0.05 FDR threshold and the fourth (astrocyte) borderline above it, indicates that lower local S1P production is associated with higher T cell tissue residency. Figure 2E places those module-level correlations alongside per-gene mean expression of the S1P-axis enzymes across eight cell-type rows (six stromal cell types plus Tumor and T cell) in control tumors. The per-gene panel shows that the production-arm enzymes (Sphk1, Sphk2, Spns2) and the degradation-arm enzyme Sgpl1 partition reciprocally across stromal cell types, anchoring the module-level couplings in panels A–D. Per-sample scatter plots for all six stromal cell types are provided in Supplementary Figure S4; beyond the four in Figure 2A–D, these add oligodendrocyte (included among the five FDR-corrected couplings above) and microglia (shown for completeness, outside the FDR family).

### 3.3. Donor-Level Stromal S1P Production Is Reduced in Human GBM Microglia

**Figure 3:**
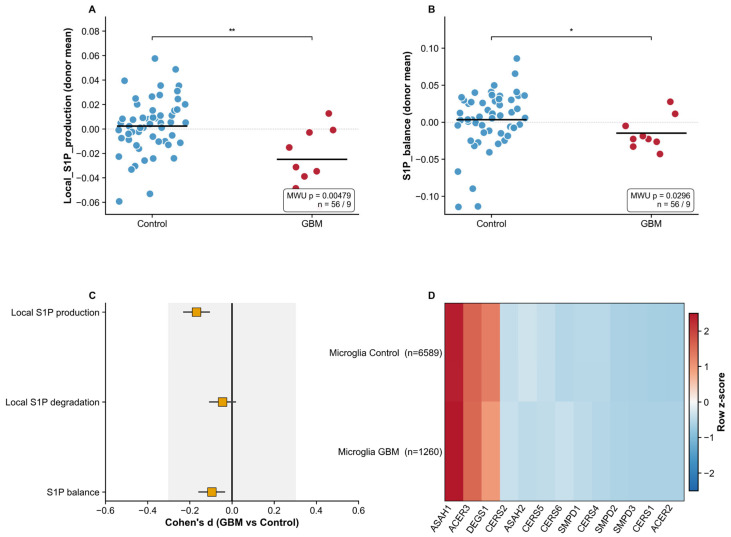
Donor-level stromal S1P production is reduced in human GBM microglia (Sankowski 2019 [35], GSE135437). (**A**) Per-donor mean local S1P production score in control vs. GBM microglia, with Mann–Whitney bracket. Donor counts and exact *p*-value annotated. (**B**) Per-donor mean S1P balance score (local production minus local degradation) in control vs. GBM microglia. (**C**) Cohort-level effect-size forest (Cohen’s d, GBM vs. control with 95% CI) for the three sphingolipid modules (local S1P production, local S1P degradation, S1P balance) computed in Sankowski microglia. Shaded gray band marks |d| < 0.3. (**D**) Microglia ceramide-axis gene heat map (mean log1p per row; row z-score) showing GBM and control rows. Box plots (**A**,**B**) show the median (center line), interquartile range (box) and whiskers, with each point being one donor (control *n* = 56, GBM *n* = 9); brackets denote two-tailed Mann–Whitney U tests on per-donor means (significance at *p* < 0.05). The forest (**C**) plots Cohen’s d (GBM vs. control) with 95% confidence intervals. Panel (**D**) is a descriptive expression map (no disease test): mean log1p-normalized expression, row z-scored. Overall, panels (**A**–**C**) show that the stromal loss of local S1P production observed in mouse GBM is reproduced at the donor level in human GBM microglia, and panel (**D**) shows a parallel broad reduction in ceramide-axis enzymes in GBM relative to control microglia. * *p* < 0.05, ** *p* < 0.01.

To test whether the stromal signal observed in mouse CT2A-derived GBM replicates in human GBM patients, we analyzed the Sankowski 2019 [35] FACS-sorted (CD45 + CD11b+) microglia scRNA-seq cohort spanning GBM and control human brains at the donor level (GSE135437; *n* = 9 GBM and *n* = 56 control donors). Because cell isolation in this cohort was restricted to the CD45 + CD11b+ microglia gate, no T cells or other stromal cell types are present in this dataset; therefore, all panels in Figure 3 reflect microglia only. Donor mean local S1P production scores (module genes: SPHK1, SPHK2, SPNS2, ABCC1, MFSD2B) were reduced in GBM microglia relative to control microglia (Figure 3A; Mann–Whitney U test on per-donor means: control *n* = 56, GBM *n* = 9 donors; *p* = 0.005), and the S1P balance composite (production module minus degradation module: SGPL1, PLPP1, PLPP2, PLPP3) showed a parallel reduction (Figure 3B; Supplementary Table S3). A cell-level Cohen’s d forest (GBM versus control microglia) places the local S1P production and S1P degradation bars on the negative side of zero, consistent with the mouse coupling result (Figure 3C). Figure 3C shows the three sphingolipid module bars only; T cell modules are omitted because the cohort contains no T cells. At the per-gene level, the ceramide-axis enzyme panel (ASAH1, ACER3, DEGS1, CERS2, ASAH2, CERS5, CERS6, SMPD1, CERS4, SMPD2, SMPD3, CERS1, ACER2) shows a broad reduction in GBM microglia relative to control microglia (Figure 3D; rows are the two microglia groups, control *n* = 6589 cells and GBM *n* = 1260 cells; values are row z-scored mean log-normalized expression). Two stromal datasets in two species therefore point in the same direction, although the human evidence is restricted to the microglia compartment.

### 3.4. Cross-Cancer Replication in CD3-Sorted GBM and Melanoma TILs

**Figure 4:**
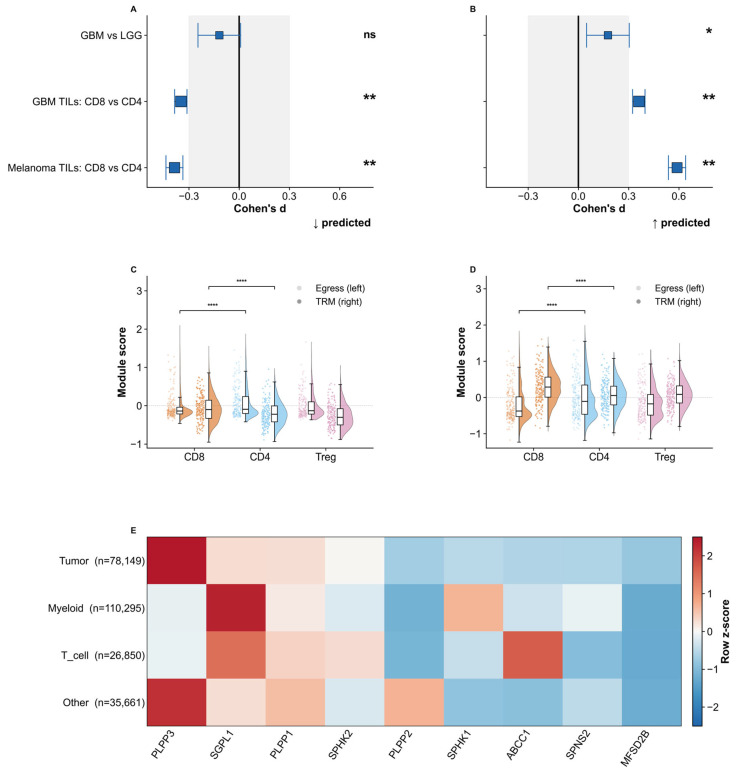
Cross-cancer replication of the T cell residency phenotype across glioblastoma and melanoma TILs, and compartment-resolved sphingolipid enzyme expression in human GBM. (**A**,**B**) Cohen’s d (95% CI) forest plots for T cell egress (predicted ↓ in tumor TILs) and T cell TRM (predicted ↑ in tumor TILs) across three cohorts (GSE182109 pan-T, Mathewson 2021 [34] GBM TIL CD8 vs. CD4, Sade-Feldman 2018 [36] melanoma TIL CD8 vs. CD4) using pruned modules and per-cell statistics. Shaded gray bands mark |d| < 0.3. (**C**) Mathewson 2021 [34]: per-cell T cell egress and TRM score distributions by T cell subset (CD8/CD4/Treg), with bracketed Mann–Whitney U comparisons between CD8 and CD4 on each module. (**D**) Sade-Feldman 2018 [36]: same layout for melanoma TILs. Within each panel (**C**,**D**), the left raincloud of each subset pair shows egress score (lighter alpha) and the right raincloud shows TRM score (darker alpha). (**E**) GSE182109 compartment-resolved S1P-axis gene heat map (rows = Tumor/Myeloid/T_cell/Other; mean log1p, row z-score). The Tumor compartment was identified by malignancy marker scoring (SOX2, EGFR, OLIG1, OLIG2, GFAP, VIM, NES, FABP7, MKI67) with a normal-neural counter-module suppressor (RBFOX3, NEFL, SYN1, SYP, MOG, MBP) applied within the source-author ‘Other’ cluster, as described in Section 2.1.2. In the rainclouds (**C**,**D**), each half-violin is a per-cell kernel density with an inset box (median, interquartile range, whiskers) and jittered per-cell points; brackets denote two-tailed Mann–Whitney U tests (CD8 vs. CD4; significance at *p* < 0.05). Forest points (**A**,**B**) are Cohen’s d with 95% confidence intervals. Panel (**E**) is a descriptive expression map (no disease test): mean log1p-normalized expression, row z-scored. Overall, panels (**A**–**D**) show that the CD8+ T cell tissue-residency phenotype is reproduced in CD3-sorted GBM TILs and in melanoma TILs, establishing it as a cross-cancer rather than GBM-specific feature, while panel (**E**) shows that, as in mouse CT2A-derived GBM, the tumor and myeloid compartments dominate S1P-axis enzyme variation in human GBM. * *p* < 0.05, ** *p* < 0.01, **** *p* < 0.0001.

To determine whether the T cell residency phenotype is specific to GBM or represents a more general property of solid-tumor TILs, we evaluated two TIL-enriched cohorts: Mathewson 2021 [34] CD3-sorted GBM TILs (already used in Section 3.1 to recover the CD8+ effect via a larger CD3-sorted cohort) and Sade-Feldman 2018 [36] melanoma TILs (16,291 cells with pre- and post-immunotherapy timepoints). The CD8+ versus CD4+ contrast in melanoma was comparable to or stronger than in GBM: T cell TRM Cohen’s d = +0.59, T cell egress d = −0.39 (Figure 4A,B). The Sade-Feldman cohort also captured anti-PD-1/anti-CTLA-4 immunotherapy as a perturbation: in CD8+ cells, post- versus pre-treatment TRM d = +0.18 and egress d = −0.19, consistent with reinforcement of the tissue-residency direction by immunotherapy. In Figure 4A,B, the three rows correspond to three contrasts: (i) GBM vs. LGG (GSE182109, pan-T cell level), (ii) GBM TILs CD8+ vs. CD4+ (Mathewson 2021 [34]), and (iii) Melanoma TILs CD8+ vs. CD4+ (Sade-Feldman 2018 [36]). The egress program (Figure 4A) is predicted to shift downward (negative d), and the TRM program (Figure 4B) is predicted to shift upward (positive d). The forest summary in Figure 4A,B places the three cohorts (GSE182109 pan-T, Mathewson 2021 [34], Sade-Feldman 2018 [36]) on a common Cohen’s d axis with 95% confidence intervals and an explicit |d| < 0.3 threshold band. Per-cell module score distributions by T cell subset are shown for each TIL cohort in Figure 4C (GBM TILs, Mathewson 2021 [34]) and Figure 4D (melanoma TILs, Sade-Feldman 2018 [36]; Supplementary Table S4) as raincloud plots: within each subset (CD8, CD4, Treg), the lighter half-violin shows the egress-module score distribution and the darker half-violin shows the TRM-module score distribution at the per-cell level. This view shows that the CD8+ versus CD4+ TRM gap is evident across the entire per-cell distribution and is not driven by a small tail of extreme-value cells. Returning to the human GBM cohort, compartment-resolved S1P-axis enzyme expression in GSE182109 (Figure 4E; rows = Tumor *n* = 78,149, Myeloid *n* = 110,295, T cell *n* = 26,850, and Other *n* = 35,661 from the re-annotation described in Section 2.1.2) shows that the tumor and myeloid compartments are the dominant sources of pathway-enzyme variation in human GBM, paralleling the cell-type partitioning observed in mouse CT2A-derived GBM (Figure 2E). The CD8+ TRM signature is therefore not restricted to GBM and is reproducible in melanoma tumors, paralleling the prognostically informative CD8+ TRM compartment identified in breast cancer [44].

## 4. Discussion

We find that stromal S1P metabolism (synthesis versus degradation) is associated with the T cell TRM phenotype in GBM tumors, and that this association is also conserved in melanoma. Together, the data support a single conclusion across cohorts and species. In both mouse CT2A-derived GBM tumors and human GBM microglia, the T cell TRM phenotype is accompanied by a loss of local stromal S1P production, a pattern consistent with, though not demonstrating, reduced T cell recirculation out of the tumor microenvironment. A complete causal demonstration would require perturbations to the S1P and ceramide arms of sphingolipid metabolism, which lie beyond the scope of the present datasets and are identified as the next step.

Confidence in this inference rests on the breadth of replication. On the stromal side, the association is preserved in mouse CT2A-derived GBM (four stromal cell types, all with sample-level Spearman ρ ≤ −0.5 against T cell TRM, *n* = 15 samples) and in human GBM microglia (Sankowski 2019 [35], donor-level reduction in local S1P production). On the T cell side, the phenotype appears in pan-T cells from human GBM (GSE182109, with Treg and CD4+ effect sizes well above |d| = 0.3 in the Recurrent vs. LGG contrast and CD8+ underpowered by the small LGG denominator), in sorted GBM TILs (Mathewson 2021 [34], CD8+ vs. CD4+ TRM d = +0.36, egress d = −0.35), and in melanoma TILs (Sade-Feldman 2018 [36], CD8+ vs. CD4+ TRM d = +0.59, egress d = −0.39). Agreement across this many cohorts, tumor types, species, and isolation strategies argues against a cohort-specific artifact (Supplementary Table S5).

One interpretive framework, which we advance strictly as a hypothesis rather than as a demonstrated mechanism, is that the egress of lymphocytes from tissue into blood and lymph normally tracks a sphingosine-1-phosphate (S1P) concentration gradient that is low in tissue and high in circulation [21,25]. Where stromal transcripts for local S1P production fall, this tissue-to-blood gradient would be expected to flatten, and the associated readout we observe is a coordinated loss of egress transcripts (S1PR1, KLF2, SELL) alongside a gain of residency markers, consistent with T cells settling into a TRM state rather than recirculating. We emphasize that our data are transcriptional and associational and cannot establish that an altered gradient retains T cells; the gradient itself was not measured.

Direct measurement of S1P-to-S1PR1 paracrine signaling was not possible in this work. S1P is a metabolite rather than a transcript, and stromal transcriptional readouts capture enzyme abundance rather than metabolite flux. The data support convergence between loss of local stromal S1P production and T cell TRM acquisition across multiple cell types and cohorts. Candidate paracrine mediators warranting follow-up include direct S1PR1 signaling and integrin-adhesion remodeling, notably ITGA4. ITGA4 is therapeutically actionable: the monoclonal antibody natalizumab, used in multiple sclerosis, targets this integrin and is supported by phase 3 evidence of efficacy in that indication [45], offering a translational handle for testing whether disrupting tissue-residency adhesion alters TIL behavior in solid tumors. Complementary entry points on the metabolic arm itself include ceramide-synthase modulation (CerS4/CerS6), which alters PD-L1 trafficking and Treg infiltration in solid tumors [30] and shapes antitumor T cell mitochondrial function in aging hosts [12]. A further entry point is pharmacological inhibition of SPHK2 by opaganib, which enhances CD8 T cell activation and shows additive activity with immunotherapy in preclinical multiple myeloma [32]. Consistent with this, recent work demonstrates that excess palmitate drives SPHK2-dependent sphingolipid and ceramide accumulation in CD8 T cells, restricting histone acetylation and chromatin accessibility and thereby impairing effector differentiation, while SPHK2 inhibition restores mitochondrial fitness and antitumor function [46]. Reciprocally, SphK1-generated S1P engages PPARγ to constrain T cell lipolysis and effector programming, such that genetic or pharmacological inhibition of SphK1 enhances antitumor T cell activity [47]. Together with ceramide-driven mitophagy in aging T cells [12], these findings frame the ceramide–S1P balance as a recurrent metabolic checkpoint on T cell antitumor function. These pharmacological tools position the ceramide–S1P axis as a tractable handle on TIL behavior in solid tumors and motivate testing them against the stromal-S1P-loss phenotype identified here. These therapeutic directions are offered as hypotheses for future experimental testing rather than as implications of the present data, which neither measures sphingolipid metabolites nor evaluates any pharmacological intervention.

The finding presented here is consistent with and complementary to the bone marrow T-cell sequestration phenotype described by Chongsathidkiet and colleagues in GBM patients [17]. That work showed that naïve T cells in patients with intracranial tumors lose surface S1PR1 expression via internalization and become trapped in the bone marrow, failing to enter the circulation in sufficient numbers to mount an antitumor response. Their findings localize the S1P-axis disruption to the T cell itself and the bone marrow compartment. Our findings localize a related disruption to the tumor site and to the stromal compartment: local S1P production by stromal cells declines, and T cells that do reach the tumor acquire a TRM phenotype characterized by loss of egress transcripts and gain of tissue-resident memory markers. Both observations implicate the S1P axis in T cell trafficking pathology in GBM, but in different anatomical compartments and at different cellular levels of the same axis. A unifying interpretation is that S1P-axis dysregulation in glioblastoma operates at multiple stations of the T cell trafficking circuit: bone-marrow naïve T cell exit failure [17] limits the pool of cells available to traffic to the tumor, and stromal S1P-production loss at the tumor site is associated with the cells that do arrive, acquiring a tissue-resident state. Whether these two manifestations are causally connected, for instance, through tumor-derived factors that propagate to distant bone marrow and disrupt the S1P gradient there, or are independent consequences of broader tumor-associated inflammation, is an open question that direct paired lipidomic and trafficking measurements would address.

Several limitations warrant explicit discussion. First, the present work is a scRNA-seq-based prediction. Module scoring reflects pathway-level transcriptional changes but does not directly measure metabolite concentrations, and no aspect of the proposed model is established here as a mechanism. Orthogonal lipidomic validation by LC-MS quantification of S1P, sphingosine, ceramides, and dihydroceramide species in tumor versus adjacent nontumor tissue is needed to verify that the transcriptional shifts we observe correspond to coordinated changes in the metabolites themselves. Second, genetic validation through cell-type-conditional knockout of sphingosine kinases in stromal compartments (for example, endothelial-Cre × Sphk1/2 flox crosses, pericyte-Cre × Sphk1/2 flox crosses, or myeloid-Cre × Sphk1/2 flox crosses) with TIL phenotyping as the readout would test whether stromal local S1P production is causally upstream of the T cell residency program rather than merely correlated with it. Third, the mouse CT2A-derived GBM cohort represents a single tumor model in a syngeneic background, and all treatment arms were analyzed together as inter-treatment controls rather than as a dedicated untreated-only cohort, which would provide a cleaner baseline for the associations reported here. Fourth, the Mathewson 2021 [34] cohort that resolved the CD8+ statistical power limitation comprised only five patients, and larger CD3-sorted cohorts would refine the effect-size estimates. Finally, transmission of the stromal signal to T cells remains a correlative observation, and identifying the operative paracrine mediator is a necessary next step. Beyond these, the cohorts analyzed here differ in species, tumor model, sequencing platform, and cell-isolation strategy, and this biological and technical heterogeneity is a potential source of confounding. We mitigated batch and platform effects by analyzing each cohort independently and requiring replication of the associations across cohorts rather than pooling cells [48], but residual dataset-specific technical and biological heterogeneity may still influence the module scores and cannot be fully excluded.

We therefore state plainly that any translational reading of these findings is speculative. The present study is transcriptomic and associational: it does not measure S1P or related sphingolipid species, does not assay S1P-to-S1PR1 signaling activity, and includes no functional or genetic perturbation. Accordingly, the inference that pharmacological modulation of the stromal S1P axis would alter T cell trafficking or antitumor immunity in glioblastoma is a hypothesis generated by these data, not a conclusion supported by them, and should not be read as a recommendation for clinical action. Direct lipidomic quantification of tumor and stromal S1P together with functional validation are prerequisites before any therapeutic implication can be entertained; until then, the stromal S1P axis is best regarded as a candidate the present associations nominate for testing.

If this model holds, stromal S1P-axis modulators warrant experimental, preclinical evaluation in GBM, and our data nominate the T cell TRM phenotype as a candidate downstream readout of impaired antitumor TIL function. Paracrine mediators of the stromal-to-T cell signal, including but not limited to ITGA4-axis adhesion molecules, represent alternative entry points for experimental investigation.

## 5. Conclusions

T cell residency in GBM tumors is accompanied by a loss of stromal-localized S1P production across four mouse stromal cell types and is reproduced in human GBM microglia at the donor level. The same T cell TRM phenotype is observed in sorted GBM TILs and melanoma TILs. Across these cohorts, lower stromal S1P production consistently accompanies higher T cell tissue residency. The convergence of two solid-tumor types on this axis motivates direct lipidomic and pharmacological evaluation of the sphingolipid pathway in GBM and other solid tumors and identifies T cell TRM as a rational downstream readout of reduced T cell recirculation and impaired antitumor function. Quantitatively, this convergence rests on consistently negative sample-level correlations between stromal local S1P production and T cell residency across four mouse stromal cell types (Spearman ρ between −0.50 and −0.67), a significant donor-level reduction in local S1P production in human GBM microglia (Mann–Whitney *p* = 0.005), and T cell tissue-residency effect sizes exceeding |d| = 0.3 in CD3-sorted GBM TILs (TRM d = +0.36, egress d = −0.35) and in melanoma TILs (TRM d = +0.59, egress d = −0.39).

This study advances the field in three principal respects. First, it relocates the S1P-axis disturbance in glioblastoma from the T cell itself to the surrounding tumor stroma, complementing the established bone-marrow naïve-T-cell sequestration phenotype by implicating a distinct anatomical station of the same trafficking axis. Second, it resolves the association at the level of individual stromal cell types—bone marrow-derived macrophage, pericyte, endothelial, and astrocyte—rather than treating the stroma as a single undifferentiated compartment. Third, by recovering the same T cell tissue-residency phenotype across five single-cell cohorts spanning two species (human and mouse), two solid-tumor types (glioblastoma and melanoma), and three cell-isolation strategies, it establishes the coupling between stromal S1P-production loss and T cell residency as a reproducible, cross-cancer feature rather than a cohort-specific observation. To our knowledge, this is the first multi-cohort, single-cell analysis to link stromal—rather than T-cell-intrinsic—loss of S1P production to the T cell TRM phenotype in glioblastoma.

The principal contribution of this work is to localize the S1P-axis disturbance to the tumor stroma rather than the T cell alone: across an integrated mouse CT2A microenvironment, transcripts for local S1P production fall in the stromal compartments (BMDM, pericyte, endothelial, and astrocyte) where the T cell TRM program rises, with a concordant donor-level reduction in human GBM microglia. These observations are associational and transcriptomic; module scores are proxies for pathway activity, and the study includes neither direct S1P metabolite measurement nor functional or genetic perturbation. The findings therefore nominate, but do not establish, stromal S1P metabolism as a candidate determinant of TIL recirculation. Definitive testing will require orthogonal LC-MS sphingolipid lipidomics, cell-type-conditional perturbation of stromal sphingosine kinases, and correlation of these signatures with clinical outcome in larger patient cohorts.

Should these associations be confirmed by direct lipidomic measurement and by stromal-specific perturbation, stromal S1P metabolism would represent a previously underappreciated and potentially actionable node for restoring T cell recirculation in immunologically cold tumors, and the T cell TRM program would provide an accessible single-cell readout for monitoring such interventions. More broadly, by reframing tumor-infiltrating lymphocyte retention as a stromal-metabolic—rather than purely T-cell-intrinsic—problem, the present work offers a concrete and experimentally testable direction for the rational design of combination strategies aimed at re-mobilizing antitumor T cells in glioblastoma and other solid tumors.

## Data Availability

All publicly available single-cell datasets analyzed in this study can be accessed under their respective Gene Expression Omnibus accessions: GSE182109, GSE163108, GSE135437, and GSE120575. The in-house mouse CT2A integrated single-cell object (all treatment arms) and per-cell module scores for all cohorts will be deposited at Zenodo prior to publication. Analysis code is available at https://github.com/chasewalton/stromal-sphingolipid-trm-axis (accessed on 4 May 2026). Per-figure result tables are provided in Supplementary Tables S1–S5.

## Supplementary Materials

The following supporting information can be downloaded at https://www.mdpi.com/article/10.3390/cancers18132168/s1, Figure S1: GSE182109 Tumor compartment re-annotation QC and final cell-type counts. The Abdelfattah 2022 ‘Other’ cluster (*n* = 113,810 cells) was subdivided into Tumor vs Other-non-tumor by scoring each cell on a malignancy marker module (SOX2, EGFR, OLIG1, OLIG2, GFAP, VIM, NES, FABP7, MKI67) together with a normal-neural counter-module (RBFOX3, NEFL, SYN1, SYP, MOG, MBP). (A) Re-annotation threshold space. Cells are placed in the malignancy-score × normal-neural-score plane and colored by the final compartment assignment; Tumor calls (red) are the cells satisfying malignancy score > 0 and normal-neural score ≤ 0 (*n* = 78,149 of 113,810). (B) Final cell-type counts in the Abdelfattah 2022 object used in main-text Figure 4E (Tumor, Myeloid, T cell, Other). This is the only re-clustering/re-labeling step performed on any public cohort in this work and is documented in the analysis repository at reannotate_gse182109_tumor.py; Figure S2: Per-cohort cell-type composition by sample for the two cohorts in which we performed per-sample analyses. (A) GSE182109 (Abdelfattah 2022 human GBM, *n* = 13 donors); stacked bars show Tumor, Myeloid, T cell, and Other fractions per donor, ordered by tumor stage (LGG, Primary, Recurrent). (B) In-house mouse CT2A control cohort (*n* = 15 samples); stacked bars show the full cell-type composition per sample (Tumor, BMDM, Microglia, Endothelial, Pericyte, Astrocyte, Oligodendrocyte, T cell, B cell, dendritic cell, neutrophil, mast cell, NK cell). This panel provides the denominator structure underlying the donor-level statistics in Figure 1 and the per-sample Spearman correlations in Figure 2 and Supplementary Figure S4; Figure S3: GSE182109 per-T-cell-subset Cohen’s d forest plot with 95% confidence intervals underlying the main-text Figure 1D heat map. (A) Primary vs LGG contrast. (B) Recurrent vs LGG contrast. Within each panel rows are T cell subsets (CD8+, CD4+, Treg, Other) × module (T cell egress shown as circles, T cell TRM as squares). Per-cell *n* is annotated for each row. The forest makes explicit the wide confidence intervals around the CD8+ contrast in the LGG comparison (driven by only 82 LGG CD8+ cells in this cohort), motivating the CD8+ statistical-power rescue using the CD3-sorted Mathewson 2021 cohort presented in Section 3.4; Figure S4: Mouse CT2A per-sample stromal–T cell TRM coupling extended to all six stromal cell types. Per-sample (*n* = 15 mouse control samples) scatter of stromal Local S1P production score against T cell TRM score for the six stromal compartments represented in the main-text Figure 2E gene-level heat map: (A) BMDM, (B) Microglia, (C) Endothelial, (D) Pericyte, (E) Astrocyte, and (F) Oligodendrocyte. BMDM, Endothelial, Pericyte, and Astrocyte are also shown in main-text Figure 2A–D; Microglia and Oligodendrocyte are additional here. Each point is one mouse sample. Dashed line is the linear regression fit, truncated at observed data bounds. Spearman ρ, two-tailed p value, and sample size are annotated per panel. This figure completes the six-stromal-cell-type coupling picture summarized in the main-text Section 3.2; Table S1: Per-T-cell-subset effect sizes for human glioblastoma (GSE182109; Figure 1). Cohen’s d (standard error, 95% CI) for the T-cell egress and tissue-resident-memory (TRM) modules, by T-cell subset and tumour-stage contrast. n_pos/n_neg are cell counts in each arm of the contrast; Table S2: Mouse CT2A stromal–T-cell coupling (Figure 2). Spearman rank correlation (ρ) between per-sample stromal local-S1P-production score and per-sample T-cell TRM score (*n* = 15 control samples), with raw p and Benjamini–Hochberg q across the five tested stromal cell types; followed by the underlying per-sample scores plotted in Figure 2A–D; Table S3: Donor-level sphingolipid module scores in human GBM microglia (Sankowski 2019, GSE135437; Figure 3). Cohort-level effect sizes (Cohen’s d, GBM vs control) for three sphingolipid modules, followed by per-donor mean scores (GBM *n* = 9; control *n* = 56); Table S4: Cross-cancer tumour-infiltrating-lymphocyte (TIL) effect sizes (Figure 4). Cohen’s d (95% CI) for the T-cell egress and TRM modules across cohorts; followed by per-subset module-score summaries for the two CD3-enriched TIL cohorts; Table S5: Cross-cohort summary of the principal effect sizes. T-cell egress and TRM module effect sizes (Cohen’s d) across the human and mouse T-cell cohorts, and the stromal local-S1P-production–to–T-cell-TRM coupling (Spearman ρ) in the mouse CT2A stroma.

## Abbreviations

The following abbreviations are used in this manuscript:

BMDM	Bone marrow-derived macrophage
CD	Cluster of differentiation
CERS	Ceramide synthase
FDR	False discovery rate
GBM	Glioblastoma, IDH-wildtype
GEO	Gene Expression Omnibus
IDH	Isocitrate dehydrogenase
ITGA4	Integrin α4
KLF2	Krüppel-like factor 2
LGG	Low-grade glioma
PD-1	Programmed cell death protein 1
PD-L1	Programmed cell death ligand 1
S1P	Sphingosine-1-phosphate
S1PR1	Sphingosine-1-phosphate receptor 1
scRNA-seq	Single-cell RNA sequencing
SELL	L-selectin (CD62L)
SGPL1	Sphingosine-1-phosphate lyase 1
SPHK1/2	Sphingosine kinase 1/2
TCR	T cell receptor
TILs	Tumor-infiltrating lymphocytes
TRM	Tissue-resident memory
