# Supplementary material for: Tumor and Stromal Sphingolipid Imbalance Are Associated with T Cell Tissue Residency in Glioblastoma
Source: Cancers (Basel). 2026 Jul 6;18(13):2168. doi: 10.3390/cancers18132168 (PMC13359813; doi:10.3390/cancers18132168)
Supplement: Supplementary file 1 [file cancers-18-02168-s001.zip › cancers-4385124-supplementary.pdf]

cancers

MDPI

Supplementary Materials

Supplementary Materials for: Tumor and Stromal Sphingolipid Imbalance Are Associated with T Cell Tissue Residency in Glioblastoma

Chase M. Walton, Elif Percin, Han Gyul Lee, Odai Darawsha, Ben A. Strickland and Besim Ogretmen

This document contains Supplementary Figures S1–S5 referenced in the main text. Supplementary Tables S1–S5 (per-figure statistical exports and cross-cohort summary tables) are provided as separate machine-readable files.

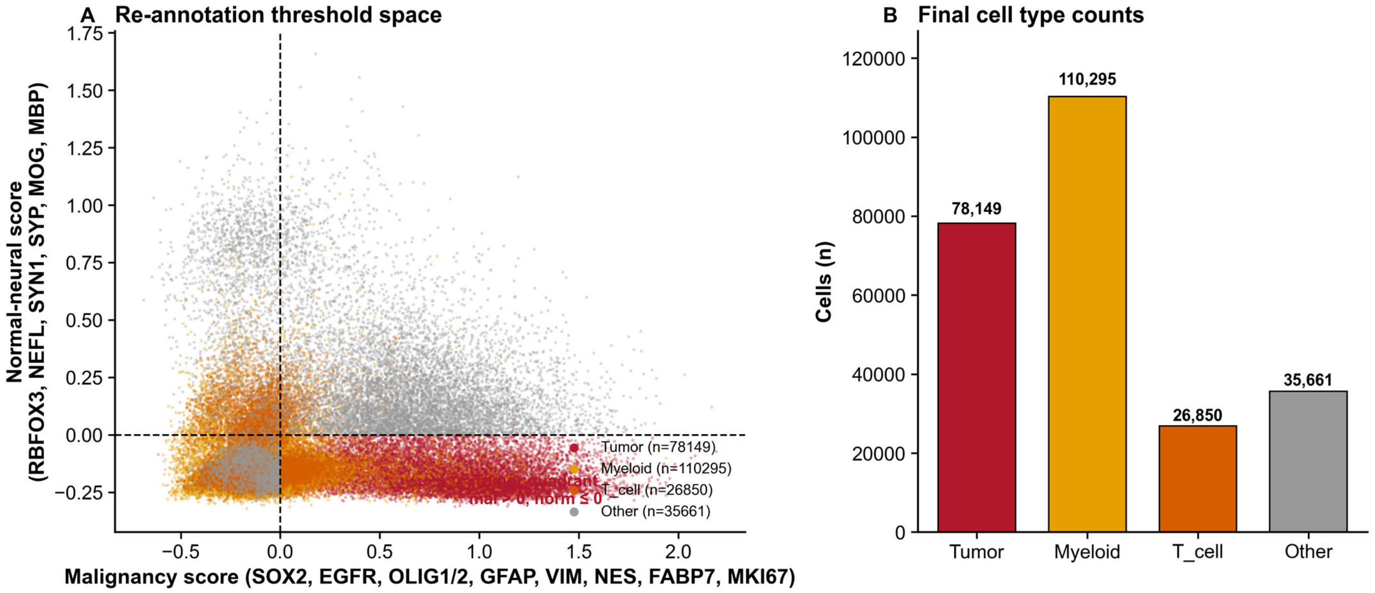

Figure S1. GSE182109 Tumor compartment re-annotation QC and final cell-type counts. The Abdelfattah 2022 'Other' cluster (n = 113,810 cells) was subdivided into Tumor vs Other-non-tumor by scoring each cell on a malignancy marker module (SOX2, EGFR, OLIG1, OLIG2, GFAP, VIM, NES, FABP7, MKI67) together with a normal-neural counter-module (RBFOX3, NEFL, SYN1, SYP, MOG, MBP). (A) Re-annotation threshold space. Cells are placed in the malignancy-score × normal-neural-score plane and colored by the final compartment assignment; Tumor calls (red) are the cells satisfying malignancy score > 0 and normal-neural score ≤ 0 (n = 78,149 of 113,810). (B) Final cell-type counts in the Abdelfattah 2022 object used in main-text Figure 4E (Tumor, Myeloid, T cell, Other). This is the only re-clustering/re-labeling step performed on any public cohort in this work and is documented in the analysis repository at reannotate_gse182109_tumor.py.

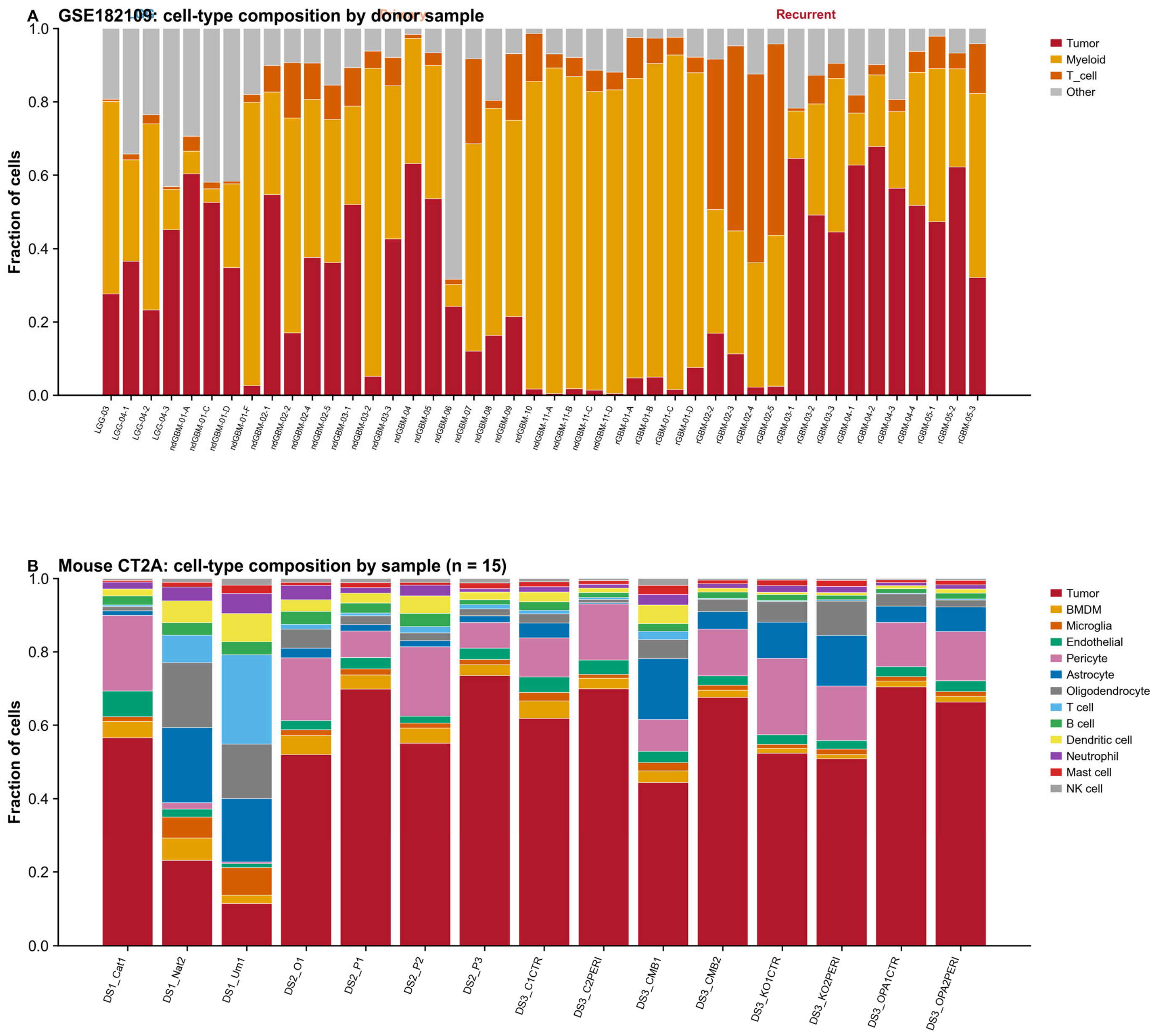

Figure S2. Per-cohort cell-type composition by sample for the two cohorts in which we performed per-sample analyses. (A) GSE182109 (Abdelfattah 2022 human GBM, $n = 13$ donors); stacked bars show Tumor, Myeloid, T cell, and Other fractions per donor, ordered by tumor stage (LGG, Primary, Recurrent). (B) In-house mouse CT2A control cohort ($n = 15$ samples); stacked bars show the full cell-type composition per sample (Tumor, BMDM, Microglia, Endothelial, Pericyte, Astrocyte, Oligodendrocyte, T cell, B cell, dendritic cell, neutrophil, mast cell, NK cell). This panel provides the denominator structure underlying the donor-level statistics in Figure 1 and the per-sample Spearman correlations in Figure 2 and Supplementary Figure S4.

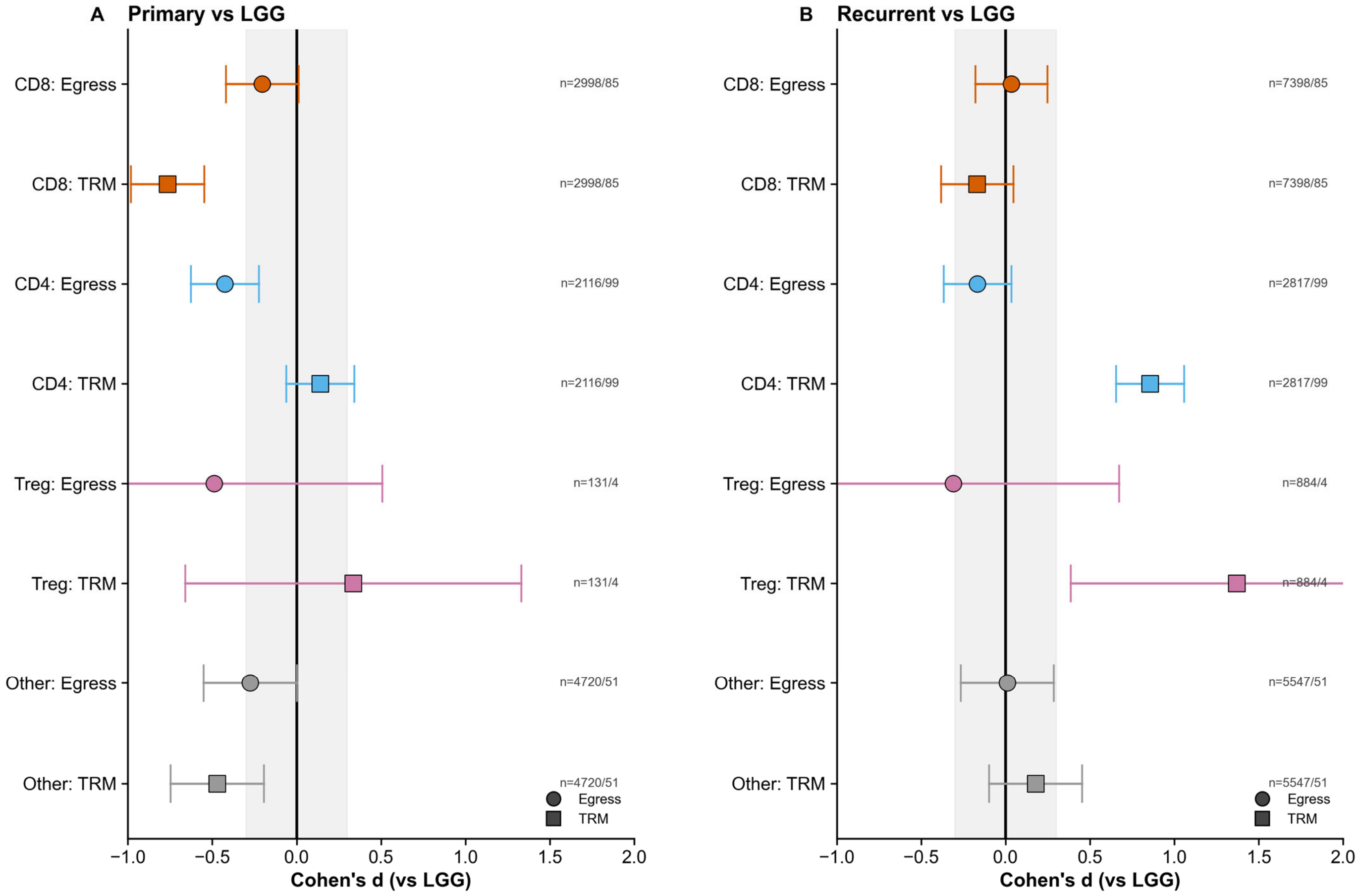

Figure S3. GSE182109 per-T-cell-subset Cohen's d forest plot with 95% confidence intervals underlying the main-text Figure 1D heat map. (A) Primary vs LGG contrast. (B) Recurrent vs LGG contrast. Within each panel rows are T cell subsets (CD8+, CD4+, Treg, Other) × module (T cell egress shown as circles, T cell TRM as squares). Per-cell *n* is annotated for each row. The forest makes explicit the wide confidence intervals around the CD8+ contrast in the LGG comparison (driven by only 82 LGG CD8+ cells in this cohort), motivating the CD8+ statistical-power rescue using the CD3-sorted Mathewson 2021 cohort presented in Section 3.4.

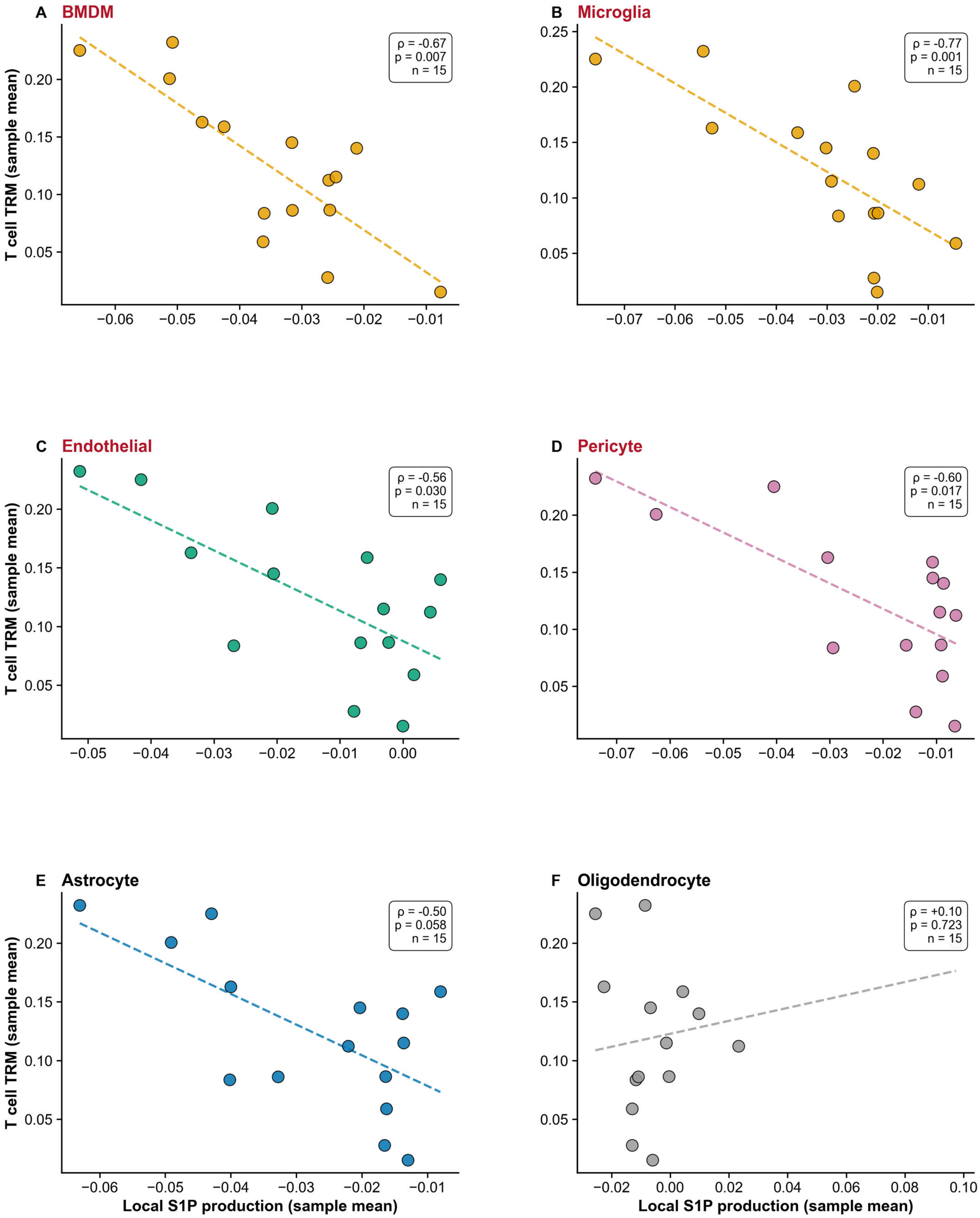

Figure S4. Mouse CT2A per-sample stromal–T cell TRM coupling extended to all six stromal cell types. Per-sample (n = 15 mouse control samples) scatter of stromal Local S1P production score against T cell TRM score for the six stromal compartments represented in the main-text Figure 2E gene-level heat map: (A) BMDM, (B) Microglia, (C) Endothelial, (D) Pericyte, (E) Astrocyte, and (F) Oligodendrocyte. BMDM, Endothelial, Pericyte, and Astrocyte are also shown in main-text Figure 2A–D; Microglia and Oligodendro-

cyte are additional here. Each point is one mouse sample. Dashed line is the linear regression fit, truncated at observed data bounds. Spearman ϱ, two-tailed p value, and sample size are annotated per panel. This figure completes the six-stromal-cell-type coupling picture summarized in the main-text Section 3.2.

Supplementary Tables

Table S1. Per-T-cell-subset effect sizes for human glioblastoma (GSE182109; Figure 1). Cohen's d (standard error, 95% CI) for the T-cell egress and tissue-resident-memory (TRM) modules, by T-cell subset and tumour-stage contrast. n_pos/n_neg are cell counts in each arm of the contrast.

Contrast	Subset	Module	Cohen's d	SE	n_pos	n_neg	CI lower	CI upper
Primary vs LGG	CD8	Egress	-0.205	0.11	2998	85	-0.421	0.01
Primary vs LGG	CD8	TRM	-0.766	0.11	2998	85	-0.982	-0.549
Primary vs LGG	CD4	Egress	-0.426	0.103	2116	99	-0.627	-0.224
Primary vs LGG	CD4	TRM	0.139	0.103	2116	99	-0.063	0.341
Primary vs LGG	Treg	Egress	-0.489	0.508	131	4	-1.486	0.507
Primary vs LGG	Treg	TRM	0.335	0.508	131	4	-0.66	1.331
Primary vs LGG	Other	Egress	-0.276	0.141	4720	51	-0.552	0
Primary vs LGG	Other	TRM	-0.472	0.141	4720	51	-0.748	-0.195
Recurrent vs LGG	CD8	Egress	0.034	0.109	7398	85	-0.18	0.248
Recurrent vs LGG	CD8	TRM	-0.169	0.109	7398	85	-0.382	0.045
Recurrent vs LGG	CD4	Egress	-0.167	0.102	2817	99	-0.367	0.034
Recurrent vs LGG	CD4	TRM	0.856	0.103	2817	99	0.654	1.057
Recurrent vs LGG	Treg	Egress	-0.31	0.501	884	4	-1.293	0.672
Recurrent vs LGG	Treg	TRM	1.37	0.502	884	4	0.385	2.354
Recurrent vs LGG	Other	Egress	0.009	0.141	5547	51	-0.266	0.285
Recurrent vs LGG	Other	TRM	0.178	0.141	5547	51	-0.098	0.454

Table S2. Mouse CT2A stromal–T-cell coupling (Figure 2). Spearman rank correlation (ϱ) between per-sample stromal local-S1P-production score and per-sample T-cell TRM score (*n* = 15 control samples), with raw p and Benjamini–Hochberg q across the five tested stromal cell types; followed by the underlying per-sample scores plotted in Figure 2A–D.

Spearman coupling: stromal local S1P production vs T-cell TRM

Stromal cell type	Spearman ϱ	p	BH q	n samples
BMDM	-0.668	0.006	0.033	15
Endothelial	-0.561	0.03	0.05	15
Pericyte	-0.604	0.017	0.043	15
Astrocyte	-0.5	0.058	0.072	15
Oligodendrocyte	0.1	0.723	0.723	15

Per-sample scores (stromal local S1P production and T-cell TRM, by stromal cell type)

Sample	BMDM local S1P production	BMDM T-cell TRM	Endothelial local S1P production	Endothelial T-cell TRM	Pericyte local S1P production	Pericyte T-cell TRM	Astrocyte local S1P production	Astrocyte T-cell TRM
DS1_Cat1	-0.036	0.084	-0.027	0.084	-0.029	0.084	-0.04	0.084
DS1_Nat2	-0.051	0.201	-0.021	0.201	-0.063	0.201	-0.049	0.201
DS1_Um1	-0.051	0.232	-0.051	0.232	-0.074	0.232	-0.063	0.232
DS2_O1	-0.046	0.163	-0.034	0.163	-0.03	0.163	-0.04	0.163
DS2_P1	-0.042	0.159	-0.006	0.159	-0.011	0.159	-0.008	0.159
DS2_P2	-0.066	0.225	-0.042	0.225	-0.041	0.225	-0.043	0.225
DS2_P3	-0.021	0.14	0.006	0.14	-0.009	0.14	-0.014	0.14
DS3_C1CTR	-0.032	0.145	-0.021	0.145	-0.011	0.145	-0.02	0.145
DS3_C2PERI	-0.026	0.112	0.004	0.112	-0.006	0.112	-0.022	0.112
DS3_CMB1	-0.032	0.086	-0.007	0.086	-0.016	0.086	-0.033	0.086
DS3_CMB2	-0.026	0.086	-0.002	0.086	-0.009	0.086	-0.016	0.086
DS3_KO1CTR	-0.008	0.015	0	0.015	-0.007	0.015	-0.013	0.015
DS3_KO2PERI	-0.026	0.028	-0.008	0.028	-0.014	0.028	-0.017	0.028
DS3_OPA1CTR	-0.024	0.115	-0.003	0.115	-0.009	0.115	-0.014	0.115
DS3_OPA2PERI	-0.036	0.059	0.002	0.059	-0.009	0.059	-0.016	0.059

Table S3. Donor-level sphingolipid module scores in human GBM microglia (Sankowski 2019, GSE135437; Figure 3). Cohort-level effect sizes (Cohen's d, GBM vs control) for three sphingolipid modules, followed by per-donor mean scores (GBM n = 9; control n = 56).

Cohort-level effect sizes

Module	Cohen's d (GBM vs Ctrl)	SE	n GBM cells	n Ctrl cells	CI lower	CI upper
Local S1P production	-0.168	0.031	1260	6589	-0.229	-0.108
Local S1P degradation	-0.045	0.031	1260	6589	-0.105	0.015
S1P balance	-0.096	0.031	1260	6589	-0.156	-0.036

Per-donor mean module scores

Donor	Group	Local S1P production	Local S1P degradation	S1P balance
GBM1_1_5	GBM	-0.015	-0.01	-0.005
GBM1_2_6	GBM	0.013	-0.015	0.028
GBM2_GBM_1_5	GBM	-0.064	-0.021	-0.043
GBM3_Pl1_1_5	GBM	-0.048	-0.015	-0.033
GBM3_Pl1_2_6	GBM	-0.035	-0.008	-0.026

Donor	Group	Local S1P production	Local S1P degradation	S1P balance
GBM3_Pl2_1_7	GBM	-0.001	-0.012	0.011
GBM3_Pl2_2_8	GBM	-0.003	0.02	-0.023
GBM4_1_7	GBM	-0.031	-0.009	-0.023
GBM4_2_8	GBM	-0.039	-0.02	-0.019
Pat10_GM_1_5	Control	0.035	0	0.036
Pat10_GM_2_6	Control	0.035	0	0.036
Pat10_WM_2_8	Control	0.005	0.012	-0.006
Pat11_Pl1_1_5	Control	-0.031	0.059	-0.09
Pat11_Pl1_2_6	Control	-0.002	0.111	-0.114
Pat11_Pl2_1_7	Control	0.031	-0.055	0.086
Pat11_Pl2_1_8	Control	-0.023	0.044	-0.067
Pat12_1_1	Control	-0.024	-0.016	-0.008
Pat12_2_2	Control	-0.005	-0.019	0.013
Pat13_GM_1_1	Control	-0.053	-0.012	-0.041
Pat13_GM_2_2	Control	-0.016	0.011	-0.027
Pat14_Plate1_1_1	Control	0.02	0.017	0.003
Pat14_Plate1_2_2	Control	0.025	-0.015	0.04
Pat14_Plate2_1_3	Control	0.008	-0.008	0.016
Pat14_Plate2_2_4	Control	0.008	0.001	0.008
Pat15_GM_1_1	Control	0.009	-0.02	0.028
Pat15_GM_2_2	Control	0.005	0	0.006
Pat15_WM_1_3	Control	0.011	-0.019	0.03
Pat15_WM_2_4	Control	0.011	-0.012	0.023
Pat1_GM_1_5	Control	0.02	-0.016	0.036
Pat1_GM_2_6	Control	0.008	0.004	0.003
Pat1_WM_1_7	Control	0.002	-0.023	0.025
Pat1_WM_2_8	Control	0.028	-0.004	0.031
Pat2_GM_1_1	Control	-0.007	-0.01	0.003
Pat2_GM_2_2	Control	0.005	-0.008	0.012
Pat2_WM_1_3	Control	-0.024	-0.034	0.01
Pat2_WM_2_4	Control	0.004	0.016	-0.011
Pat3_GM_1_1	Control	-0.011	-0.008	-0.003
Pat3_GM_2_2	Control	0.005	-0.004	0.009
Pat3_WM_1_3	Control	0.009	-0.002	0.012
Pat3_WM_2_4	Control	0.026	-0.014	0.041
Pat4_GM_1_5	Control	-0.03	0.002	-0.032
Pat4_GM_2_6	Control	-0.059	0.055	-0.114

Donor	Group	Local S1P production	Local S1P degradation	S1P balance
Pat4_WM_1_11	Control	-0.033	-0.008	-0.025
Pat4_WM_1_12	Control	-0.01	0.015	-0.025
Pat5_GM_1_1	Control	-0.002	-0.03	0.027
Pat5_GM_2_2	Control	0.001	-0.025	0.026
Pat5_WM_1_3	Control	-0.001	0.003	-0.004
Pat5_WM_2_4	Control	0.015	0.023	-0.008
Pat6_Plate1_1_1	Control	-0.009	-0.01	0.001
Pat6_Plate1_2_2	Control	-0.013	-0.012	-0.001
Pat6_Plate2_1_5	Control	0.024	0.007	0.018
Pat6_Plate2_1_6	Control	-0.006	-0.011	0.005
Pat7_GM_2_2	Control	0.049	-0.017	0.066
Pat7_WM2_MS_Ctrl_1_7	Control	0.001	0.016	-0.015
Pat7_WM2_MS_Ctrl_2_8	Control	-0.013	0.005	-0.019
Pat7_WM_1_3	Control	0.039	0.01	0.03
Pat7_WM_2_4	Control	0.058	0.008	0.05
Pat8_GM_1_1	Control	0.015	-0.021	0.037
Pat8_GM_2_2	Control	0.016	-0.025	0.041
Pat8_WM_1_3	Control	0.009	0.013	-0.004
Pat8_WM_2_4	Control	-0.01	0.019	-0.029
Pat9_GM_1_1	Control	0.008	-0.025	0.034
Pat9_GM_2_2	Control	-0.026	-0.012	-0.014
Pat9_WM_1_5	Control	0.001	-0.006	0.007
Pat9_WM_2_6	Control	-0.004	-0.008	0.004

Table S4. Cross-cancer tumour-infiltrating-lymphocyte (TIL) effect sizes (Figure 4). Cohen's d (95% CI) for the T-cell egress and TRM modules across cohorts; followed by per-subset module-score summaries for the two CD3-enriched TIL cohorts.

Effect sizes (Figure 4A,B)

Cohort / contrast	Module	Cohen's d	CI lower	CI upper	n_pos	n_neg
GSE182109 (pan-T, GBM vs LGG)	Egress	-0.119	-0.246	0.009	26611	239
GSE182109 (pan-T, GBM vs LGG)	TRM	0.177	0.049	0.304	26611	239
Mathewson 2021 (GBM TIL, CD8 vs CD4)	Egress	-0.348	-0.385	-0.311	21502	3277

Cohort / contrast	Module	Cohen's d	CI lower	CI upper	n_pos	n_neg
Mathewson 2021 (GBM TIL, CD8 vs CD4)	TRM	0.361	0.324	0.398	21502	3277
Sade-Feldman 2018 (melanoma TIL, CD8 vs CD4)	Egress	-0.386	-0.437	-0.335	6797	1940
Sade-Feldman 2018 (melanoma TIL, CD8 vs CD4)	TRM	0.589	0.538	0.64	6797	1940

Per-subset module-score summaries (Figure 4C,D)

Cohort	Subset	Module	n cells	Mean	Median	SD
Mathewson 2021	CD8	Egress	21502	-0.044	-0.141	0.318
Mathewson 2021	CD8	TRM	21502	-0.075	-0.102	0.349
Mathewson 2021	CD4	Egress	3277	0.071	-0.095	0.392
Mathewson 2021	CD4	TRM	3277	-0.199	-0.222	0.303
Mathewson 2021	Treg	Egress	388	-0.007	-0.13	0.319
Mathewson 2021	Treg	TRM	388	-0.269	-0.304	0.309
SadeFeldman 2018	CD8	Egress	6797	-0.209	-0.375	0.476
SadeFeldman 2018	CD8	TRM	6797	0.291	0.285	0.403
SadeFeldman 2018	CD4	Egress	1940	-0.02	-0.109	0.541
SadeFeldman 2018	CD4	TRM	1940	0.057	0.055	0.374
SadeFeldman 2018	Treg	Egress	833	-0.151	-0.176	0.415
SadeFeldman 2018	Treg	TRM	833	0.086	0.085	0.344

Table S5. Cross-cohort summary of the principal effect sizes. T-cell egress and TRM module effect sizes (Cohen's d) across the human and mouse T-cell cohorts, and the stromal local-S1P-production–to–T-cell-TRM coupling (Spearman ρ) in the mouse CT2A stroma.

T-cell module effect sizes (egress predicted ↓, TRM predicted ↑ in tumour)

Cohort / contrast	Egress Cohen's d	TRM Cohen's d
GSE182109 (pan-T, GBM vs LGG)	-0.119	0.177
Mathewson 2021 (GBM TIL, CD8 vs CD4)	-0.348	0.361
Sade-Feldman 2018 (melanoma TIL, CD8 vs CD4)	-0.386	0.589

Human GBM microglia, Sankowski 2019 (Figure 3)

Sphingolipid module	Cohen's d (GBM vs Control microglia)
Local S1P production	-0.168
Local S1P degradation	-0.045
S1P balance	-0.096

Mouse CT2A stromal coupling: local S1P production vs T-cell TRM (n = 15; Figure 2)

Stromal cell type	Spearman ρ	p	BH q
BMDM	-0.668	0.006	0.033
Endothelial	-0.561	0.03	0.05
Pericyte	-0.604	0.017	0.043
Astrocyte	-0.5	0.058	0.072
Oligodendrocyte	0.1	0.723	0.723
